# *In vitro* Relative Fitness, *in vivo* Intestinal Colonization and Genomic Differences of *Escherichia coli* of ST131 Carrying *bla*_CTX–M–15_

**DOI:** 10.3389/fmicb.2021.798473

**Published:** 2022-02-18

**Authors:** Frederik Boëtius Hertz, Rasmus L. Marvig, Niels Frimodt-Møller, Karen Leth Nielsen

**Affiliations:** ^1^Department of Clinical Microbiology, Rigshospitalet, Copenhagen, Denmark; ^2^Department of Genomic Medicine, Rigshospitalet, Copenhagen, Denmark

**Keywords:** ESBL, WGS, whole-genome sequencing, urinary tract infection, beta-lactamase, MLST typing, intestinal colonization, comparative genomics

## Abstract

**Introduction:**

Extended-spectrum beta-lactamase (ESBL) producing *Escherichia coli* have become prevalent worldwide, with *E. coli* of sequence type 131 (ST131) as the dominant genotype. *E. coli* ST131 predominantly exhibits the serotype O25, is associated with the ESBL CTX-M-15 and belongs to a well-defined subclade within the Fim*H*30-R clade, Fim*H*30-Rx/C2. Multidrug resistance may have fitness costs for the bacteria. The aim of the current study was to investigate the fitness burden compared to a susceptible ST131 isolate without resistance genes *in vitro* and *in vivo* and describe genetic differences between fit and less fit isolates.

**Materials and methods:**

From a collection of clinical ESBL and non-ESBL *E. coli* isolates from urinary tract infection, we selected 16 *bla*_CTX–M–15_-positive isolates of ST131. The *in vitro* fitness was examined, and relative bacterial fitness (fit_*t*_) was determined by direct competition with a fully susceptible ST131 isolate and illustrated in percent, with <100% resulting in a lower fitness, compared to the susceptible reference isolate. The isolates were subjected to whole-genome sequencing and analyzed for resistance markers, plasmids, phage content, and serotype. *In vivo* competition was tested in a mouse colonization model.

**Results:**

The majority (12 out of 16) of the CTX-M-15-producing isolates had a slightly lower relative fitness compared to the susceptible ST131 isolate (mean, 97.6%; range, 82.6–108%) *in vitro*. Three isolates had a better fitness than the susceptible ST131 isolate, and one isolate had an identical fitness to the susceptible ST131 isolate. The *in vitro* fitness showed no correlation to the number of plasmids, number of phages, number of resistances, or genome size. For the *in vivo* competition assays, all three ESBL-producing isolates showed better colonization of the ESBL-resistant ST131 isolates compared to the susceptible ST131 isolate.

**Conclusion:**

This study shows that ESBL-producing ST131/*H*30-Rx are not necessarily burdened by multidrug resistance, however, have a better *in vitro* fitness than the susceptible isolate. These data contribute to the understanding of the success of ST131/*H*30-Rx, although they do not indicate ways to overcome this highly fit, virulent, and antimicrobial-resistant clone.

## Introduction

Extended-spectrum beta-lactamase (ESBL) producing *Escherichia coli* have become prevalent worldwide, with *E. coli* of sequence type 131 (ST131) as the dominant genotype (Boll et al., 2013). *E. coli* ST131 predominantly exhibits the serotype O25 and is commonly associated with the ESBL CTX-M-15 (Boll et al., 2013). The vast majority of ST131 isolates carrying *bla*_CTX–M–15_ belongs to a well-defined clade within the Fim*H*30-R cluster, Fim*H*30-Rx/C2, exhibiting multidrug resistance (MDR) (Price et al., 2013). *H*30 has a close association with allele 30 of the type 1 fimbrial adhesin FimH, which mediates colonization and invasion of the bladder epithelium, facilitates the formation of biofilm, mediates binding to the intestinal crypts, and assists in the establishment of a stable gastrointestinal reservoir (Boll et al., 2013; Klein and Hultgren, 2020). Finally, *E. coli* causes a wide range of infections, including urinary tract and bloodstream infections and in patients presenting to emergency departments with sepsis, of which approximately 27% of cases can be attributed to “urosepsis” (Klein and Hultgren, 2020). The evolution of the ST131/*H*30 clone has evolved from acquisition of virulence-associated genes followed by the development of antibiotic resistance, and these events have driven its expansion as a world dominant clone (Ben Zakour et al., 2016).

Multidrug resistance often has a cost for the bacteria and consequently hinders the possibility to survive in a competitive environment. Fitness of a bacteria is multifaceted, and a part from fast growth rate, good fitness can also be achieved by e.g., good colonization abilities, ability to adapt to available nutrients or by having an increased virulence and specific genetic content (Hejnova et al., 2005; Leatham-Jensen et al., 2016). Multidrug-resistant clone with poor fitness has previously been shown to be outcompeted by faster growing clones with a lower resistance burden if the antimicrobial consumption, and hence, selection pressure, is lowered (Nielsen et al., 2012). Carriage of ESBL has been described by Schaufler et al. (2016) to not lead to a fitness loss in itself for the bacteria. One study of the fitness of a single isolate belonging to ST131/*H*30 showed similar results (Johnson et al., 2016). A recent study compared MDR *E. coli* ST131 clade B to clade C, which emerged from clade B, and oppositely found that clade C isolates of the worldwide expanding clone had lower *in vivo* fitness than clade B isolates (Duprilot et al., 2020). Fitness studies on a larger collection of ST131/*H*30 isolates has, to our knowledge, not been performed.

The aim of the current study was to investigate whether CTX-M-15-producing *E. coli* of ST131 had a fitness burden compared to an ST131 isolate without resistance genes and whether the fitness of the isolates could be linked to specific genetic markers or genetic relationship. This was investigated with *in vitro* competition assays in correlation to the genomic analyses and in a mouse colonization model where *in vivo* competition was performed.

## Materials and Methods

### Bacterial Isolates

From a collection of clinical ESBL and non-ESBL *E. coli* isolates from urinary tract infection in general practice, Zealand, Denmark (Hertz et al., 2016), we selected 16 *bla*_CTX–M–15_-positive isolates of ST131 belonging to O-antigen O25 (*n* = 11), O16 (*n* = 3), O153 (*n* = 1) and without O-antigen (*n* = 1), respectively. The isolates were selected to represent the various O-types within ST131 all carrying bla_CTX–M–15_.

### *In vitro* Competition

The *in vitro* fitness was examined as previously described by Nielsen et al. (2012). We competed each of the 16 ST131 isolates carrying ESBL against a fully susceptible ST131 isolate from the same collection of clinical isolates (Table 1). Briefly, the susceptible and one ESBL isolate were mixed 1:1 in LB propagating the cultures by daily transfer to fresh medium over 3 days, counting the number of ampicillin-resistant and ampicillin-susceptible colonies each day. Selective plating was performed on LB and LB + 100 μg/ml ampicillin agar plates in order to distinguish the growth of the two isolates. Each competition assay was performed in duplicates of up to four competition cycles and serial dilutions were plated in duplicate. The relative fitness of the isolates was calculated as previously described (Sander et al., 2002; Nielsen et al., 2012). Briefly, relative bacterial fitness (fit_*t*_) is defined by Sander et al. (2002) as fit_*t*_ = 1 + S_*t*_, where S_*t*_ is calculated as:


$$S_{t}=l\,n\,\left[{\left(\frac{r_{t}/s_{t}}{r_{t-1}/s_{t-1}}\right)}^{1/18}\right]$$

**TABLE 1:** Isolate characteristics and relative fitness when compared to susceptible ST131 isolate (Hvi138).

Isolate	Fit t%	±SE %	*N*	*p*-value*	Serotype (O)	*fim*H
Hvi130	97.3	2.56	6	0.12	O25	*H*30
Hvi41	108	0.03	7	0.11	O25	*H*30
Hvi09	98.5	0.01	12	0.04	O25	*H*30
Hvi77	98.0	2.79	6	0.33	N/A	*H*30
Hvi100	92.7	2.77	6	0.02	O25	*H*30
Hvi66	98.3	0.80	6	0.09	O25	*H*30
Hvi80	104	0.87	6	0.5	O25	*H*30
Hvi45	82.6	2.26	2	0.08	O25	*H*30
Hvi31	100	0.01	12	1	O25	*H*30
Hvi59	99.8	0.44	6	0.8	O25	*H*30
Hvi39	92.6	1.98	12	0.0043	O25	*H*30
Hvi132	98.3	1.07	6	0.08	O25	*H*35
Hvi49	105	0.03	6	0.2	O153	*H*41
Hvi123	93.2	0.99	6	0.001	O16	*H*41
Hvi23	94.1	0.74	12	<0.0001	O16	*H*41
Hvi78	95.7	1.60	6	0.0075	O16	*H*41

Fit_t_ %, relative bacterial fitness; n, number of fit_t_, in which the average fit_t_ was calculated from; SE, standard error mean (%). *p-value calculated by comparing fit_t_ to zero (fitness of reference isolate) by one sample t-test.

where r_*t*_ and s_*t*_ are the number of drug-resistant and drug-susceptible cells, respectively, at a given time t, and r_*t*–1_ and s_*t*–1_ are the number of drug-resistant and drug-susceptible cells, respectively, at the preceding timepoint. The quotient of the ratios of the cell numbers was standardized with the exponent 1/18 because cell numbers were determined approximately every 18th generation. The data are presented as relative bacterial fitness (fit_*t*_), defined by Sander et al. (2002). A fit_*t*_ of 1 represents identical competitive fitness to the reference isolate, whereas a fit_*t*_ < 1 indicates decreased competitive fitness compared with the reference isolate. Illustrated in percent, 100% represents identical competitive fitness to the reference isolate, whereas fit_*t*_% < 100 indicates decreased competitive fitness compared with the reference isolate.

### Whole-Genome Sequencing, Assembly, and Annotation

The isolates were subjected to whole-genome sequencing using both paired-end libraries and mate-pair libraries, in order to create high-quality genomes, especially with respect to the mobilome. The isolates were run on Illumina Miseq 2000 2 × 250 bp (500 cycles) after library preparation with Nextera XT (paired-end libraries) and Nextera Mate Pair libraries (Illumina), respectively. The genomes were assembled with Allpaths-LG with the following settings: scaffolding, insert size paired-end 300 ± 200 and 3,000 ± 1,000 for mate pair. The sequencing and following assembly yielded high-quality genomes with low scaffold counts (Supplementary Table 1). Genomes were annotated with Prokka v 1.12.

### Comparative Genome Analyses

We analyzed the genomes for resistance markers (ResFinder), plasmids (PlasmidFinder), phage content (PHAST database), and serotype (SeroTypeFinder). For phylogenetic inference, we used BacDist (Gabrielaite et al., 2020) with *E. coli* ST131 CP006784 as reference in order to create a maximum likelihood tree. We analyzed accessory genome content of three closely related isolates belonging to *H*30-cluster using GenAPI (Gabrielaite and Marvig, 2019). Accessory genome differences were visualized in Geneious Prime v2019.1.2.

### *In vivo* Competition in Mouse Intestine

We applied a streptomycin-treated mouse model, in order to reduce a large part of the fecal flora incl. *Enterobacterales* and other aerobes (Leónidas Cardoso et al., 2020) before oral inoculation of a mix 1:1 with 10^6^ CFU/ml of each isolate using a steel probe. We followed the protocol for streptomycin treatment of mice as described by Vimont et al. (2012). Briefly, in two separate experiments, mice were treated with streptomycin (3.5 and 5 g/L, respectively) in the drinking water for 5 days, followed by 5 days with normal drinking water, in order to clear the streptomycin from the mice. On the day of inoculation, feces was collected to control that no *E. coli* was present at this time. The mice were inoculated through a stainless steel orogastric feeding tube, and feces was collected on day 0 (inoculation), 1, 2, 4, and 8. Subsequently, 0.5 g of feces was soaked in 5 ml 0.9% saline for 1 h and vortexed vigorously. A 10-fold serial dilution (10^–1^–10^–6^) was created and 20 μl spotted on to chromogenic UTI brilliance agar plates in duplicates. All plates contained 5 μg/ml vancomycin and with or without ampicillin (100 μg/ml). After 24 h incubation the *E. coli* CFUs were counted on chromogenic agar (Brilliance UTI agar, Oxoid, Hampshire, United Kingdom).

### Statistics

*T*-test (*p* = 0.05) for the slope parameter was applied to differences in fitness of isolates in correlation to complete phage content. *t*-test was performed to test whether the fitness of the isolates differed significantly from the reference isolate by making a one sample *t*-test comparing fit_*t*_ to 0.

## Results and Discussion

### Relative Fitness of Sequence Type 131 Isolates

**FIGURE 1:**
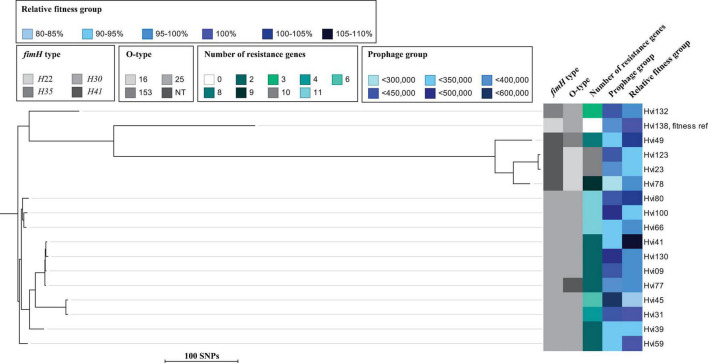
Maximum-likelihood phylogenetic tree based on core SNPs of isolates tested for *in vitro* relative fitness. Illustrated is *fimH* type, O-type, number of resistance genes, prophage content (bp), and relative fitness in percent (fit_*t*_ %). Maximum SNP distance across the tree is 880 SNPs.

Of the 16 CTX-M-15-producing isolates, 12 had a slightly lower relative fitness compared to the susceptible ST131 isolate (mean, 97.4%; range, 82.6–108%) (Table 1). The three isolates belonging to the O16/*H*41 group had a low fitness overall (mean, 94.4%; range, 93.2–95.7%) compared to the susceptible ST131 isolate, although not statistically significant (*p* = 0.22). The relative fitness of O25/*H*30 isolates was overall highly diverse (mean, 97.6%; range, 82.6–108%), despite that several of these isolates were closely related in the phylogeny (Figure 1). One example is Hvi31 (fit_*t*_ % = 100%) and Hvi45 (fit_*t*_ % = 82.6%), which are different by only 58 single-nucleotide polymorphisms (SNPs). Three isolates had a better fitness than the susceptible ST131 isolate, and one isolate had an identical fitness to the susceptible ST131 isolate (Table 1).

### Divergent Fitness Within the O25/*H*30 Cluster

**FIGURE 2:**
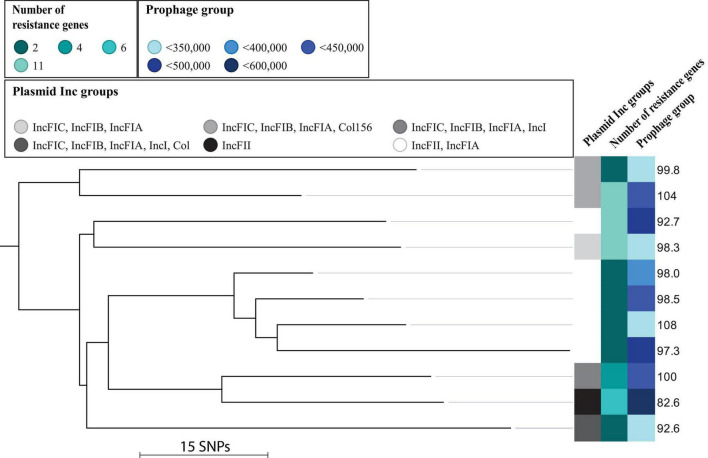
Maximum-likelihood phylogenetic tree based on core SNPs of isolates belonging to the *H*30 cluster. Illustrated is the relative fitness in percent along with plasmid Inc groups, number of resistance genes, and prophage content in bp. Maximum SNP distance across the tree is 120 SNPs.

A phylogenetic analysis of subclade ST131/O25/*H*30 isolates revealed no separate clustering of isolates with relatively poor or good fitness (Figure 2). We also observed that the relative fitness of the isolates did not correlate to the number of plasmid Inc groups or number of resistance genes (Figure 2). There was a trend with some of the isolates having a combination of good fitness and little amount of prophage-related material (e.g., isolate Hvi41), and isolates with poor fitness had large amounts of prophage-related material (e.g., Hvi45) (Figure 2). There was, however, not a significant linear correlation between the amount of complete prophage-related material and fitness across the collection in total (*t*-test for slope, *p* = 0.5). Six out of nine (67%) isolates with average or less than average total prophage material had good fitness (good fitness defined as average fitness across the collection or better). Likewise, four out of eight (50%) of isolates with more than average prophage-related material also had a poor fitness (poor fitness defined as worse than average). Similarly, we observed that the number of resistance genes was not correlated to fitness of the isolates: Hvi66 and Hvi80 carried 11 resistance genes and had a similar or better fitness than the susceptible ST131 isolate (Figure 2).

These results encouraged us to look for differences in accessory genome content for these isolates, in order to elucidate if differences in the gene repertoire could explain differences in relative fitness. We selected three isolates with different fitness and a relatively low number of SNPs between them, namely, Hvi80, Hvi41, and Hvi100, which differed from Hvi41 (fit_*t*_% = 108% fit) with 75 SNPs (Hvi80, fit_*t*_% = 103.8%) and 67 SNPs (Hvi100, fit_*t*_% = 92.7%), respectively, to represent divergent relative fitness (Figure 2). Inc groups of these isolates were identical for Hvi100 and Hvi41 with IncFII and IncFIA, whereas Hvi80 had more plasmid material represented by two additional Inc groups: IncFIB and Col156. Despite having two more Inc groups, Hvi80 had the best fitness of the three isolates, so plasmid content did not seem to affect the fitness of this particular isolate directly, which is in line with the missing trend between plasmid Inc-group content and relative fitness of the individual isolates for both O25/*H*30 and the complete ST131 cluster, which was investigated (Figures 1, 2).

The analysis revealed large variation in accessory genome content of these three isolates. The isolates differed by 142–279 genes when compared pairwise. Of the genes in each of the investigated isolates, 288–550 (6–11%) were of varying presence/absence. This represents a large genetic diversity within the ST131/*H*30 clade despite that these isolates are closely related in a core genome phylogeny with a maximum of 75 SNPs.

For Hvi41 (fit_*t*_% = 107.8), the isolate with the largest relative fitness, and hence, fastest growth rate, the varying gene content constitutes a complete phage that was not found in isolates Hvi100 and Hvi80; Phage_yersin_L_413C_NC_004745(25) and sporadic phage genes across contig 1 and 2 of the assembled genome belonging to phage_entero_BP_4795_NC_004813. The carriage of this phage could contribute to the fitness of the isolate under the specific growth conditions, as some prophages have previously been associated to increased fitness and metabolic growth advantages (Frazão et al., 2019; Wendling et al., 2021).

In Hvi80 (fit_*t*_% = 103.8), the varying gene content constitutes two phages similar to phage_entero_Sf101_NC_02739 (63,200 bp) and Phage_salmon_SSU5_NC_018843 (63,400 bp) and an IncFIB plasmid containing prophage material and resistance determinants such as *sul2*, *dfrA17*, *aac(3)-iid*, *aph(3″)-ib*, and *aph(6)-Id*, and the largest genome, yet the isolate still has one of the highest relative fitness in this collection of ST131/*H*30 isolates. This illustrates that the number of mobile genetic elements solely does not determine the fitness of the individual isolates, rather the nature of these elements, and possibly how they are genetically anchored. In addition, transcriptional levels of these genes can also contribute to their fitness cost. This has not been studied here and is a limitation of the current study.

For Hvi100 (fit_*t*_% = 92.7), the varying gene content constitutes plasmid and phage genes, which are anchored in the chromosome and two complete prophages: Phage_entero_Sf101_NC_027398 and Phage_salmon_SSU5_NC018843. The extra genetic features likely burden the bacteria and contribute to the quite large loss of fitness that this isolate endure.

The genetic diversity between these isolates represent 6–11% of the complete genome (Table 2). This is a relatively large proportion of the genome, and this major genetic difference is not illustrated in the core genome SNP phylogeny where the isolates differ with maximum 75 SNPs. This illustrates that core genome typing is not always enough to describe genetic relationships and elucidate transmission of a clone.

**TABLE 2:** Pangenome analyses of ST131/*H*30 isolates representing number of additional coding in each of the three isolates (listed in rows) and the sum of additional coding genes.

Isolate	Number of coding genes	Extra coding genes			Sum
		Hvi41	Hvi80	Hvi100	
Hvi100	5,027	233	279	−	512 (10%)
Hvi80	5,095	271	−	279	550 (11%)
Hvi41	4,974	−	146	142	288 (6%)

The genetic analyses combined with the fitness of the isolates indicate that the specific phage content could be correlated to fitness of the isolates *in vitro*. Isolates belonging to ST131/O16 had a lower mean fitness than the isolates of ST131/O25/*H*30, which could be one of the explanations for lower expansion and success of this clone compared to ST131/*H*30. The *in vitro* fitness showed no correlation to number of plasmids, number of phages, number of resistances, or genome size, rather was suspected to vary with content of mobile genetic elements.

### *In vivo* Competition

**FIGURE 3:**
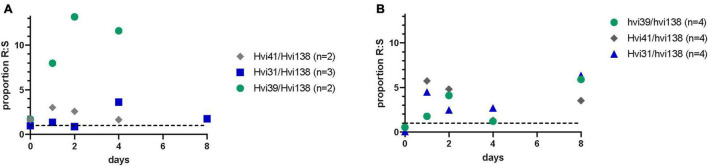
**(A)** *In vivo* proportion of CFU/ml count between resistant and susceptible strain after low dose of streptomycin and **(B)** after high dose of streptomycin. Hvi31 had an *in vitro* relative fitness of 100%, Hvi41 had a relative fitness of 108% relative fitness, and Hvi39 had a relative fitness of 92.6%. Number of cages (n) illustrates the number of caches that the proportion was based on.

For the *in vivo* competition assays, we selected the susceptible ST131 isolate from the *in vitro* fitness assay to compete with three different isolates with varying *in vitro* fitness: Hvi31 with a 100% relative fitness, Hvi41 with a 107% relative fitness, and Hvi39 with a relative fitness of 92.6%. The proportion of each isolate was counted on day 0 (inoculum), 2, 4, and 8 after inoculation. The experiment was performed with a low and high dose of streptomycin treatment prior to inoculation. The results show that the colonization duration with ST131 was dependent on the disturbance of the intestinal colonization barrier, with poor colonization in mice that received a lower concentration of streptomycin (Figure 3).

In both experiments and for all three ESBL-producing isolates, we observed better colonization of the ESBL-resistant ST131 isolates compared to the susceptible ST131 isolate—with two- to sixfold difference in proportion of CFU/ml after 8 days (Figure 3), irrespective of the relative *in vitro* fitness. This illustrates that the susceptible isolate is a poor colonizer of the mouse intestine compared to the ESBL-producing ST131 isolates, and hence, that the ESBL-producing isolates are relatively good colonizers regardless of their *in vitro* relative fitness. Noteworthy, the isolates with similar or lower *in vitro* fitness show good colonization abilities and have a higher *in vivo* proportion relative to the susceptible isolate. This illustrates the complexity in *in vivo* colonization, which is not only dependent on growth rate but also on available nutrients, virulence, and colonization resistance.

These data illustrate that the isolates do not seem to be burdened by the antimicrobial multidrug resistance with lower colonization as a result. As studies on human and mouse gastrointestinal microbiota have correlated the composition of the microbiota to possible colonization with resistant bacteria, good colonizers of ESBL-producing ST131 isolates will have an advantage in an environment containing antibiotics (Hertz et al., 2020). Furthermore, one of the most dramatic modifications to the gut microbiota is caused by antibiotic treatment, due to the disruption of the colonization barrier or colonization resistance. Thus, antibiotic treatment can cause selection of drug-resistant bacteria, such as ST131. When ST131 show good abilities to colonize the gut, this may result in a subsequent long-term colonization and possible infection caused by the ST131 (Hertz et al., 2020). We speculate whether colonization with ST131/O25/*H*30 may drive a durable carrier stage.

## Conclusion

Isolates belonging to ST131/O25/*H*30 had a varying fitness independent of *bla*_CTX–M–15_ carriage, and several of the isolates had a better fitness than the susceptible ST131 isolate despite multidrug resistance. Isolates belonging to ST131/O16/*H*41 generally had a lower fitness than the susceptible isolate, which could indicate that this clone generally has lower surviving abilities compared to the susceptible and ST131/O25/*H*30-Rx with multidrug resistance in a selective environment. The results of the present study illustrate that although previous multidrug resistant clones have been possible to eliminate by lowering the antibiotic consumption, due to a fitness loss, this may not be the case for ST131/*H*30-Rx. The present data illustrate that the isolates have a relatively good fitness despite being multidrug resistant, in addition to being relatively good colonizers in the mouse intestine, relative to a fully susceptible ST131 isolate. The accessory genome showed large variation, which could be attributed in the fitness of the isolates. These data contribute to the understanding of the success of ST131/*H*30-Rx, although they do not indicate ways to overcome this highly fit, virulent, and antimicrobial-resistant clone.

## Data Availability Statement

The sequencing data from this study are available in SRA under accession number PRJNA790005. The data behind the animal experiments can be found in Supplementary Data Sheet 1.

## Ethics Statement

The animal study was reviewed and approved by Danish Animal Ethics Council, København K, Denmark.

## Author Contributions

FH, NF-M, and KN: study design. KN, RM, and FH: experimental work. KN, RM, FH, and NF-M: data analysis and final manuscript. FH and KN: writing first draft. NF-M: funding acquisition. All authors contributed to the article and approved the submitted version.

## Conflict of Interest

The authors declare that the research was conducted in the absence of any commercial or financial relationships that could be construed as a potential conflict of interest.

## Publisher’s Note

All claims expressed in this article are solely those of the authors and do not necessarily represent those of their affiliated organizations, or those of the publisher, the editors and the reviewers. Any product that may be evaluated in this article, or claim that may be made by its manufacturer, is not guaranteed or endorsed by the publisher.
