# Supplementary material for: In vitro Relative Fitness, in vivo Intestinal Colonization and Genomic Differences of Escherichia coli of ST131 Carrying blaCTX–M–15
Source: Front Microbiol. 2022 Feb 18;12:798473. doi: 10.3389/fmicb.2021.798473 (PMC8894762; doi:10.3389/fmicb.2021.798473)
Supplement: Supplementary file 2 [file Table_1.DOCX]

Supplementary table S1

| Isolate ID | Number of scaffolds | Genome size |
| --- | --- | --- |
| Hvi130 | 18 | 5,438,961 |
| Hvi41 | 20 | 5,488,388 |
| Hvi09 | 17 | 5,430,325 |
| Hvi77 | 30 | 5,361,762 |
| Hvi100 | 30 | 5,516,939 |
| Hvi66 | 23 | 5,372,464 |
| Hvi80 | 24 | 5,637,303 |
| Hvi45 | 14 | 5,433,456 |
| Hvi31 | 17 | 5,776,990 |
| Hvi59 | 17 | 5,156,877 |
| Hvi39 | 21 | 5,399,484 |
| Hvi132 | 28 | 5,426,699 |
| Hvi49 | 26 | 5,428,172 |
| Hvi123 | 17 | 5,493,043 |
| Hvi23 | 25 | 5,616,329 |
| Hvi78 | 16 | 5,311,890 |
| Hvi138 | 17 | 5,306,147 |
